# Supplementary material for: Clinical manifestations of bed bug bites: A systematic review of case reports
Source: PLoS One. 2026 Apr 29;21(4):e0341398. doi: 10.1371/journal.pone.0341398 (PMC13127920; doi:10.1371/journal.pone.0341398)
Supplement: S1 File — (DOCX) [file pone.0341398.s001.docx]

**Supplement 2. Comparison of symptomatology and bite location according to age and sex**

| Variable | Children (0-17) | Young Adults (18-39) | Middle-aged Adults (40-64) | Older Adults (65+) | p_value | p_Bon | p_BY |
| --- | --- | --- | --- | --- | --- | --- | --- |
| Face | 6 (60.0) | 6 (18.8) | 2 (7.4) | 2 (16.7) | 0.005 | 0.345 | 0.554 |
| Ear Canal | 0 (0.0) | 1 (3.1) | 0 (0.0) | 0 (0.0) | 0.671 | 1.000 | 1.000 |
| Scalp | 0 (0.0) | 0 (0.0) | 1 (3.7) | 1 (8.3) | 0.400 | 1.000 | 1.000 |
| Eyelids | 5 (50.0) | 0 (0.0) | 0 (0.0) | 0 (0.0) | <0.001 | 0.007 | 0.017 |
| Hands | 0 (0.0) | 3 (9.4) | 0 (0.0) | 1 (8.3) | 0.313 | 1.000 | 1.000 |
| Neck | 3 (30.0) | 7 (21.9) | 1 (3.7) | 2 (16.7) | 0.151 | 1.000 | 1.000 |
| Buttocks | 1 (10.0) | 4 (12.5) | 0 (0.0) | 6 (50.0) | <0.001 | 0.007 | 0.017 |
| Inguinal | 0 (0.0) | 2 (6.2) | 1 (3.7) | 2 (16.7) | 0.358 | 1.000 | 1.000 |
| Arms | 7 (70.0) | 21 (65.6) | 21 (77.8) | 9 (75.0) | 0.765 | 1.000 | 1.000 |
| Legs | 7 (70.0) | 18 (56.2) | 15 (55.6) | 8 (66.7) | 0.795 | 1.000 | 1.000 |
| Abdomen | 1 (10.0) | 6 (18.8) | 3 (11.1) | 5 (41.7) | 0.126 | 1.000 | 1.000 |
| Trunk | 7 (70.0) | 6 (18.8) | 10 (37.0) | 5 (41.7) | 0.024 | 1.000 | 1.000 |
| Back | 0 (0.0) | 4 (12.5) | 6 (22.2) | 6 (50.0) | 0.014 | 0.966 | 1.000 |
| Exanthem | 10 (100.0) | 25 (78.1) | 21 (77.8) | 11 (91.7) | 0.296 | 1.000 | 1.000 |
| Paleness | 0 (0.0) | 0 (0.0) | 1 (3.7) | 0 (0.0) | 0.567 | 1.000 | 1.000 |
| Crusts | 0 (0.0) | 2 (6.2) | 2 (7.4) | 0 (0.0) | 0.657 | 1.000 | 1.000 |
| Cough | 0 (0.0) | 1 (3.1) | 1 (3.7) | 0 (0.0) | 0.853 | 1.000 | 1.000 |
| Dyspnea | 0 (0.0) | 1 (3.1) | 0 (0.0) | 0 (0.0) | 0.671 | 1.000 | 1.000 |
| Excoriations | 0 (0.0) | 1 (3.1) | 1 (3.7) | 1 (8.3) | 0.768 | 1.000 | 1.000 |
| Necrosis | 0 (0.0) | 0 (0.0) | 1 (3.7) | 0 (0.0) | 0.567 | 1.000 | 1.000 |
| Swollen orbit | 1 (10.0) | 1 (3.1) | 0 (0.0) | 0 (0.0) | 0.334 | 1.000 | 1.000 |
| Vasculitis | 0 (0.0) | 1 (3.1) | 2 (7.4) | 1 (8.3) | 0.708 | 1.000 | 1.000 |
| Rosacea | 0 (0.0) | 1 (3.1) | 0 (0.0) | 0 (0.0) | 0.671 | 1.000 | 1.000 |
| Telangiectasia | 0 (0.0) | 0 (0.0) | 0 (0.0) | 1 (8.3) | 0.121 | 1.000 | 1.000 |
| Impetigo | 0 (0.0) | 1 (3.1) | 0 (0.0) | 0 (0.0) | 0.671 | 1.000 | 1.000 |
| Otitis | 0 (0.0) | 1 (3.1) | 0 (0.0) | 0 (0.0) | 0.671 | 1.000 | 1.000 |
| Anemia | 0 (0.0) | 0 (0.0) | 4 (14.8) | 3 (25.0) | 0.025 | 1.000 | 1.000 |
| Fatigue | 0 (0.0) | 2 (6.2) | 1 (3.7) | 1 (8.3) | 0.797 | 1.000 | 1.000 |
| Tachycardia | 0 (0.0) | 1 (3.1) | 1 (3.7) | 1 (8.3) | 0.768 | 1.000 | 1.000 |
| Dizziness | 0 (0.0) | 2 (6.2) | 1 (3.7) | 2 (16.7) | 0.358 | 1.000 | 1.000 |
| Lethargy | 0 (0.0) | 2 (6.2) | 1 (3.7) | 1 (8.3) | 0.797 | 1.000 | 1.000 |
| Flares | 0 (0.0) | 1 (3.1) | 0 (0.0) | 0 (0.0) | 0.671 | 1.000 | 1.000 |
| Fever | 0 (0.0) | 0 (0.0) | 1 (3.7) | 1 (8.3) | 0.400 | 1.000 | 1.000 |
| Hyperpigmentation | 0 (0.0) | 2 (6.2) | 0 (0.0) | 0 (0.0) | 0.371 | 1.000 | 1.000 |
| Malaise | 0 (0.0) | 0 (0.0) | 0 (0.0) | 1 (8.3) | 0.121 | 1.000 | 1.000 |
| Synovitis | 0 (0.0) | 0 (0.0) | 0 (0.0) | 1 (8.3) | 0.121 | 1.000 | 1.000 |
| Arthralgia | 0 (0.0) | 0 (0.0) | 0 (0.0) | 1 (8.3) | 0.121 | 1.000 | 1.000 |
| Edema | 0 (0.0) | 0 (0.0) | 0 (0.0) | 1 (8.3) | 0.121 | 1.000 | 1.000 |
| Conjunctivitis | 0 (0.0) | 0 (0.0) | 0 (0.0) | 1 (8.3) | 0.121 | 1.000 | 1.000 |
| Palpitations | 0 (0.0) | 1 (3.1) | 0 (0.0) | 0 (0.0) | 0.671 | 1.000 | 1.000 |
| Anorexia | 0 (0.0) | 0 (0.0) | 1 (3.7) | 0 (0.0) | 0.567 | 1.000 | 1.000 |
| Weight Loss2 | 0 (0.0) | 1 (3.1) | 1 (3.7) | 0 (0.0) | 0.853 | 1.000 | 1.000 |
| Linear lesions | 2 (20.0) | 16 (50.0) | 15 (55.6) | 4 (33.3) | 0.195 | 1.000 | 1.000 |
| Exanthem: Targetoid | 0 (0.0) | 1 (3.1) | 0 (0.0) | 0 (0.0) | 0.671 | 1.000 | 1.000 |
| Exanthem: Blister | 2 (20.0) | 5 (15.6) | 2 (7.4) | 0 (0.0) | 0.343 | 1.000 | 1.000 |
| Exanthem: Papular | 10 (100.0) | 24 (75.0) | 17 (63.0) | 10 (83.3) | 0.116 | 1.000 | 1.000 |
| Exanthem: Bullous | 0 (0.0) | 7 (21.9) | 4 (14.8) | 1 (8.3) | 0.334 | 1.000 | 1.000 |
| Exanthem: Macula | 8 (80.0) | 15 (46.9) | 16 (59.3) | 5 (41.7) | 0.220 | 1.000 | 1.000 |
| Exanthem: Petechial Plaques | 0 (0.0) | 0 (0.0) | 0 (0.0) | 1 (8.3) | 0.121 | 1.000 | 1.000 |
| Exanthem: Hemorrhagic | 0 (0.0) | 4 (12.5) | 3 (11.1) | 1 (8.3) | 0.700 | 1.000 | 1.000 |
| Pruritus | 2 (20.0) | 13 (40.6) | 9 (33.3) | 6 (50.0) | 0.625 | 1.000 | 1.000 |
| Anxiety | 0 (0.0) | 5 (15.6) | 2 (7.4) | 0 (0.0) | 0.250 | 1.000 | 1.000 |
| Sleep deprivation | 0 (0.0) | 4 (12.5) | 1 (3.7) | 0 (0.0) | 0.268 | 1.000 | 1.000 |
| Anger | 0 (0.0) | 3 (9.4) | 1 (3.7) | 1 (8.3) | 0.658 | 1.000 | 1.000 |
| Stress | 0 (0.0) | 4 (12.5) | 4 (14.8) | 0 (0.0) | 0.334 | 1.000 | 1.000 |
| Incredulity | 0 (0.0) | 1 (3.1) | 0 (0.0) | 1 (8.3) | 0.439 | 1.000 | 1.000 |
| Embarrassment | 0 (0.0) | 0 (0.0) | 0 (0.0) | 1 (8.3) | 0.121 | 1.000 | 1.000 |
| Financial distress | 0 (0.0) | 1 (3.1) | 0 (0.0) | 0 (0.0) | 0.671 | 1.000 | 1.000 |
| Hopelessness | 0 (0.0) | 5 (15.6) | 2 (7.4) | 1 (8.3) | 0.475 | 1.000 | 1.000 |
| Depression | 0 (0.0) | 4 (12.5) | 0 (0.0) | 0 (0.0) | 0.092 | 1.000 | 1.000 |
| Alcohol use | 0 (0.0) | 1 (3.1) | 1 (3.7) | 0 (0.0) | 0.853 | 1.000 | 1.000 |
| Suicide ideation | 0 (0.0) | 1 (3.1) | 1 (3.7) | 0 (0.0) | 0.853 | 1.000 | 1.000 |
| Suicide attempt | 0 (0.0) | 1 (3.1) | 1 (3.7) | 0 (0.0) | 0.853 | 1.000 | 1.000 |
| Mood lability | 0 (0.0) | 2 (6.2) | 0 (0.0) | 0 (0.0) | 0.371 | 1.000 | 1.000 |
| Social isolation | 0 (0.0) | 3 (9.4) | 4 (14.8) | 1 (8.3) | 0.598 | 1.000 | 1.000 |
| Stigma | 0 (0.0) | 3 (9.4) | 1 (3.7) | 1 (8.3) | 0.658 | 1.000 | 1.000 |
| Entomophobia | 0 (0.0) | 2 (6.2) | 1 (3.7) | 0 (0.0) | 0.699 | 1.000 | 1.000 |
| Paranoia | 0 (0.0) | 2 (6.2) | 0 (0.0) | 0 (0.0) | 0.371 | 1.000 | 1.000 |
| Carelessness | 0 (0.0) | 1 (3.1) | 3 (11.1) | 1 (8.3) | 0.493 | 1.000 | 1.000 |
